# An Antimitotic and Antivascular Agent BPR0L075 Overcomes Multidrug Resistance and Induces Mitotic Catastrophe in Paclitaxel-Resistant Ovarian Cancer Cells

**DOI:** 10.1371/journal.pone.0065686

**Published:** 2013-06-06

**Authors:** Xiaolei Wang, Erxi Wu, Jun Wu, Tian-Li Wang, Hsing-Pang Hsieh, Xinli Liu

**Affiliations:** 1 Department of Pharmaceutical Sciences, School of Pharmacy, Texas Tech University Health Sciences Center, Amarillo, Texas, United States of America; 2 Department of Pharmaceutical Sciences, North Dakota State University, Fargo, North Dakota, United States of America; 3 Division of Comparative Medicine, Beckman Research Institute of the City of Hope, Duarte, California, United States of America; 4 Departments of Gynecology/Obstetrics and Oncology, Johns Hopkins University, Baltimore, Maryland, United States of America; 5 Institute of Biotechnology and Pharmaceutical Research, National Health Research Institutes, Taipei, Taiwan, Republic of China; Cedars-Sinai Medical Center, United States of America

## Abstract

Paclitaxel plays a major role in the treatment of ovarian cancer; however, resistance to paclitaxel is frequently observed. Thus, new therapy that can overcome paclitaxel resistance will be of significant clinical importance. We evaluated antiproliferative effects of an antimitotic and antivascular agent BPR0L075 in paclitaxel-resistant ovarian cancer cells. BPR0L075 displays potent and broad-spectrum cytotoxicity at low nanomolar concentrations (IC_50_ = 2–7 nM) against both parental ovarian cancer cells (OVCAR-3, SKOV-3, and A2780-1A9) and paclitaxel-resistant sublines (OVCAR-3-TR, SKOV-3-TR, 1A9-PTX10), regardless of the expression levels of the multidrug resistance transporter P-gp and class III β-tubulin or mutation of β-tubulin. BPR0L075 blocks cell cycle at the G2/M phase in paclitaxel-resistant cells while equal concentration of paclitaxel treatment was ineffective. BPR0L075 induces cell death by a dual mechanism in parental and paclitaxel-resistant ovarian cancer cells. In the parental cells (OVCAR-3 and SKOV-3), BPR0L075 induced apoptosis, evidenced by poly(ADP-ribose) polymerase (PARP) cleavage and DNA ladder formation. BPR0L075 induced cell death in paclitaxel-resistant ovarian cancer cells (OVCAR-3-TR and SKOV-3-TR) is primarily due to mitotic catastrophe, evidenced by formation of giant, multinucleated cells and absence of PARP cleavage. Immunoblotting analysis shows that BPR0L075 treatment induced up-regulation of cyclin B1, BubR1, MPM-2, and survivin protein levels and Bcl-XL phosphorylation in parental cells; however, in resistant cells, the endogenous expressions of BubR1 and survivin were depleted, BPR0L075 treatment failed to induce MPM-2 expression and phosphorylation of Bcl-XL. BPR0L075 induced cell death in both parental and paclitaxel-resistant ovarian cancer cells proceed through caspase-3 independent mechanisms. In conclusion, BPR0L075 displays potent cytotoxic effects in ovarian cancer cells with a potential to overcome paclitaxel resistance by bypassing efflux transporters and inducing mitotic catastrophe. BPR0L075 represents a novel microtubule therapeutic to overcome multidrug resistance and trigger alternative cell death by mitotic catastrophe in ovarian cancer cells that are apoptosis-resistant.

## Introduction

Ovarian cancer, the most lethal malignancy of the gynecologic cancer, results annually in over 14,000 U.S. and 114,000 worldwide deaths. Despite advances in the diagnosis and treatment, the five-year survival rate for stage IV patients is about 18% [1], [2]. The inability to overcome drug resistance and inhibit metastasis represents the major cause of treatment failure [3]. Innovative and effective new therapeutics that overcome drug resistance are critically needed to improve the survival and quality of life of patients with this disease.

Microtubule-stabilizing agents such as taxanes, epothilones, and microtubule-destabilizing agents such as *vinca* alkaloids are among the most effective chemotherapeutics used in the clinic [4]. However, one of the biggest hurdles evolved in the clinic is multidrug resistance (MDR). Especially for paclitaxel, despite significant initial response for advanced ovarian cancer using paclitaxel and cisplatin based combination therapy, the vast majority of patients relapse and develop drug-resistance [5], [6]. Paclitaxel resistance is multifactorial, including up-regulation of membrane drug efflux transporter P-glycoprotein (P-gp) [7], [8], mutations in β-tubulin gene [9], [10], [11], [12], alterations in the expression of β-tubulin isotypes [13], [14], aberrant signal transduction pathways [15], [16], and changes in apoptotic regulatory proteins such as Bcl-2 [17], [18] and inhibitor of apoptosis protein survivin [19]. The identification of novel antimitotic agent that can overcome taxane resistance, display endurable activity in taxane-refractory tumors could potentially bring clinical benefits to patients with advanced ovarian cancer.

BPR0L075 [6-methoxy-3-(3′,4′,5′-trimethoxy-benzoyl)-1H-indole] is a novel synthetic indole compound that inhibits tubulin polymerization through binding to the colchicine-binding site of tubulin [20]. BPR0L075 is structurally related to the classical tubulin-binding and vascular disrupting agent combretastatin. BPR0L075 has shown antimitotic and antiangiogenic activity *in vitro* and *in vivo* [20], [21]. We reported that BPR0L075 displayed vascular disrupting activity by inducing rapid, albeit, temporary tumor vascular shutdown and leading to reduction of tumor perfusion in orthotopic human breast cancer xenografts [22]. BPR0L075 arrests human cervical carcinoma KB cells at the G2/M mitotic checkpoint, and induces cell apoptosis (IC_50_ = 3.6 nM) by perturbing mitochondrial membrane potential and activating the caspase-3 cascade [20]. BPR0L075 possesses good selectivity between normal and cancer cells, with IC_50_ value in normal fibroblast Detroit 551 cells higher than 1 µM [20]. BPR0L075 exhibits single agent antitumor activity against the growth of human gastric and cervical carcinoma xenografts [20]. It also synergistically enhances antitumor activity against human lung, colorectal, and cervical tumor xenografts when combined with cisplatin [21].

In the current study, we observed that BPR0L075 was highly active in paclitaxel-resistant ovarian cancer cells and their parental cells with IC_50_ values at single digit low nanomolar concentrations. BPR0L075 induced apoptosis in parental ovarian cancer cells. In contrast, it induced mitotic catastrophe in the paclitaxel-resistant ovarian cancer cells evidenced by formation of large, multinucleated polyploid cells. BPR0L075 represents a novel and promising microtubule therapeutic to overcome taxane resistance and trigger alternative cell death by mitotic catastrophe in cells that are apoptosis-resistant.

## Results

### BPR0L075 Displays Potent Cytotoxicity in Paclitaxel-resistant Human Ovarian Carcinoma Cells

**Figure 1:**
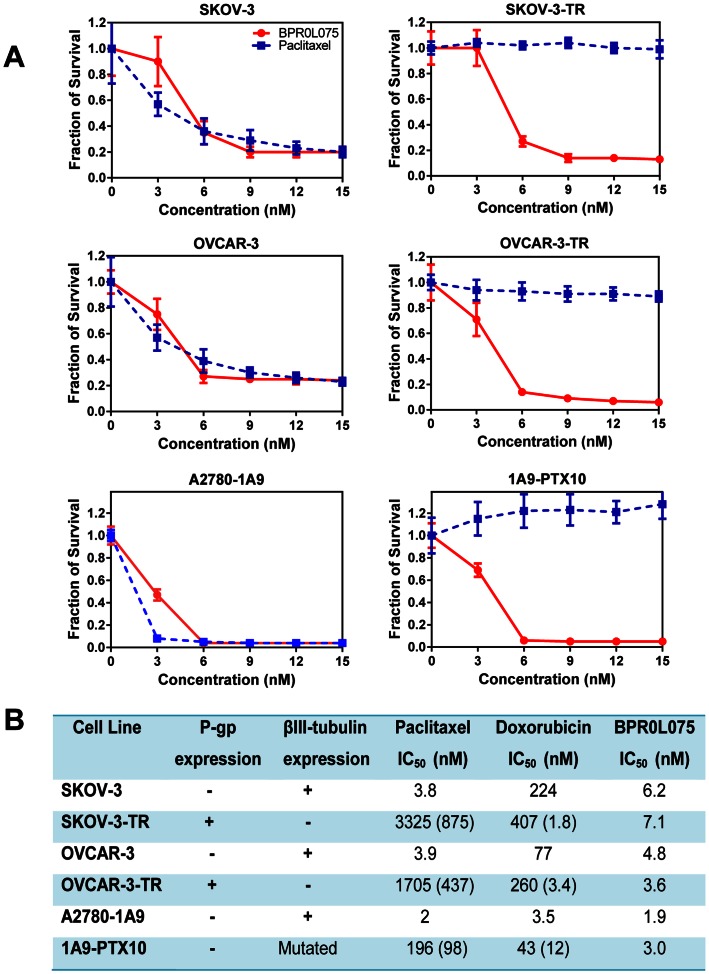
Cytotoxicity of BPR0L075 in parental and paclitaxel-resistant ovarian cancer cells. (A) Proliferating human ovarian cancer cells (SKOV-3, OVCAR-3, and A2780-1A9) and paclitaxel-resistant sublines (SKOV-3-TR, OVCAR-3-TR, and 1A9-PTX10) were treated with BPR0L075 (red circle) or paclitaxel (blue square) at 3–15 nM concentrations for 4 days and the cytotoxicity was evaluated by SRB assay. Fraction of cell survival relative to controls represents mean ± SD of 12 determinations. (B) Summary of the P-gp expression, βIII-tubulin expression, and sensitivity of paired ovarian cancer cell lines to paclitaxel, doxorubicin, and BPR0L075 treatment. Numbers in parenthesis represents the degree of resistance (x-fold), expressed as the ratio of the IC_50_ values as compared with the corresponding parental lines. All experiments were repeated three times.

We tested the cytotoxicity of BPR0L075 in human ovarian cancer cell lines SKOV-3, OVCAR-3, and A2780-1A9 as well as the paclitaxel-resistant sublines SKOV-3-TR, OVCAR-3-TR, and 1A9-PTX10, which were selected under continuous exposure of 0.25 µM paclitaxel (SKOV-3-TR), 0.5 µM paclitaxel (OVCAR-3-TR), and 15 ng/mL paclitaxel and 5 mg/mL verapamil (1A9-PTX10), respectively. Figure 1A shows that, like paclitaxel, BPR0L075 exhibited comparable antineoplastic activity in killing parental ovarian cancer cells at low nanomolar concentrations. However, BPR0L075 was more effective than paclitaxel against paclitaxel-resistant ovarian cancer cells, almost completely retaining its cytotoxic potency against these resistant sublines, where paclitaxel was virtually ineffective. Figure 1B summarizes the IC_50_ values of BPR0L075, paclitaxel, and doxorubicin against the growth of these human ovarian cancer cells and their resistant sublines after 4 days of continuous exposure. The SKOV-3-TR and OVCAR-3-TR cells showed high resistance ratios (>400-fold) for paclitaxel compared with the corresponding parental cells. 1A9-PTX10 cells displayed moderate resistance ratio (98-fold) for paclitaxel. These paclitaxel-resistant cells also showed cross-resistance to doxorubicin (1.8–12-fold). BPR0L075 displayed similar sensitivity in all paired parental and paclitaxel-resistant ovarian cell lines with IC_50_ values ranging from 2–7 nM (Figure 1B).

### BPR0L075 is not a Substrate for Efflux Transporters, and Active in Ovarian Cancer Cells with Altered βIII-tubulin Expressions and β-tubulin Mutation

**Figure 2:**
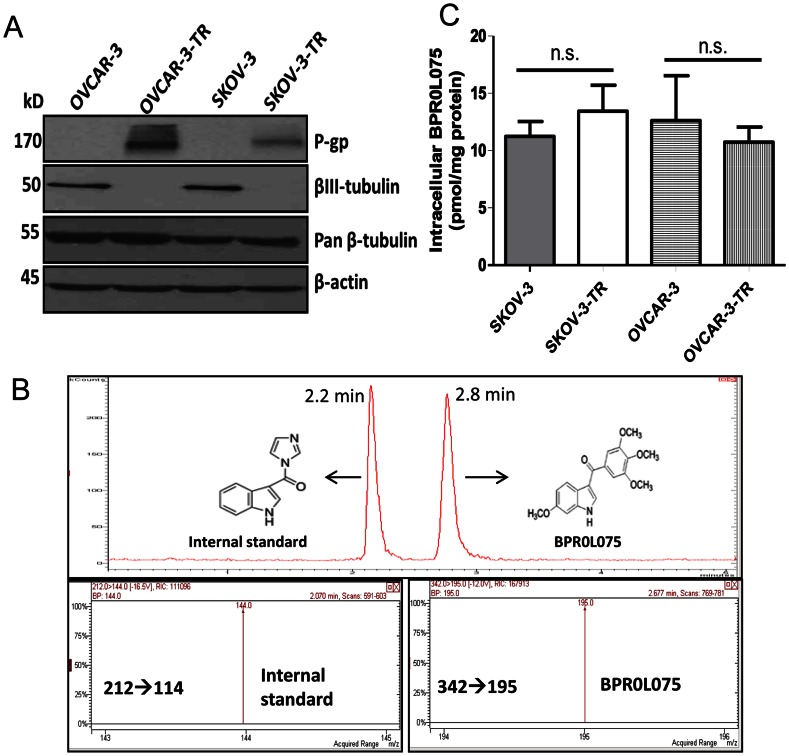
BPR0L075 is not a substrate for efflux transporters. (A) Western blot analysis of the P-gp and βIII-tubulin expressions in the paired ovarian cancer cell lines. Pan β-tubulin and β-actin were loading controls. (B) LC/MS/MS analysis of intracellular BPR0L075 levels, with LC chromatogram showing the retention time of internal standard [1-(1H-indol-3-ylcarbonyl)-1H-imidazole] and BPR0L075 as 2.2 and 2.8 min, respectively. The mass transitions of m/z 212→144 for internal standard and m/z 342→195 for BPR0L075 were monitored for quantification. (C) The intracellular BPR0L075 levels in the paired ovarian cancer cell lines. Cells were incubated with 20 nM BPR0L075 for 6 hours, then cells were washed, harvested, and lysed for LC/MS/MS measurement. Bars represent mean ± SD of three independent tests. No statistically significant difference was observed (*P*>0.05, student *t*-test).

Immunoblotting analysis showed that the resistant cells OVCAR-3-TR and SKOV-3-TR have high expression of P-gp protein while the parental OVCAR-3 and SKOV-3 cells have no detectable P-gp expression (Figure 2A). To confirm that BPR0L075 is not a substrate for efflux pumps, we measured the intracellular BPR0L075 levels in the two pairs of cell lines OVCAR-3/OVCAR-3-TR and SKOV-3/SKOV-3-TR using a sensitive LC/MS/MS method (Figure 2B). Both pairs of cells obtained similar intracellular BPR0L075 concentrations after BPR0L075 treatment (20 nM for 6 hours, Figure 2C) with no statistical difference (*P*>0.05). BPR0L075 is not a substrate for another ATP-binding cassette efflux transporter breast cancer resistance protein (BCRP), evidenced by the comparable cytotoxicity in human breast cancer MCF7 cells (IC_50_ = 3.5 nM) and mitoxantrone-resistant MCF7/MX cells (IC_50_ = 4.0 nM), MCF7/MX cells are known to overexpress BCRP [23], [24], [25]. Together, the results show that efflux transporter P-gp contributed to the selected paclitaxel resistance in SKOV-3-TR and OVCAR-3-TR cells, but BPR0L075 is not a substrate for efflux pumps, which contributes to its ability to overcome paclitaxel resistance.

Paclitaxel resistance phenotype is frequently associated with alteration of the class III β-tubulin isotype expression [26]. We observed differential expressions of tubulin-isotype βIII-tubulin in parental and paclitaxel-resistant ovarian cancer cells. Both OVCAR-3 and SKOV-3 cells have high expressions of βIII-tubulin, but after the selection with paclitaxel, the expression of βIII-tubulin markedly diminished (Figure 2A), the alteration of the βIII-tubulin expressions in resistant cells did not affect the sensitivity to BPR0L075. The 1A9-PTX10 cells are known to exhibit a non-MDR1 resistant phenotype, they harbor β-tubulin mutation with impaired paclitaxel interaction with tubulin [27], [28], [29], [30] (Figure 1B). The mutation of β-tubulin did not negatively affect the BPR0L075 induced cytotoxicity in 1A9-PTX10 cells (Figure 1). Together, BPR0L075 is cytotoxic to cells with high P-gp and BCRP expressions; the cytotoxicity is independent of βIII-tubulin expression levels or β-tubulin mutation status.

### BPR0L075 Induces G2/M Arrest in Both Parental and Paclitaxel-resistant Ovarian Cancer Cells

**Figure 3:**
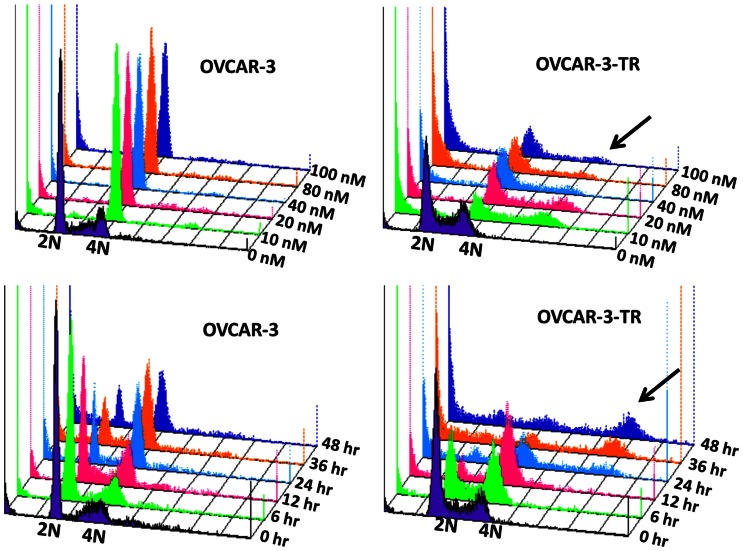
BPR0L075 induces concentration- and time-dependent cell cycle arrest in both parental and paclitaxel-resistant ovarian cancer cells. OVCAR-3 and OVCAR-3-TR cells were treated with ethanol vehicle or BPR0L075 (10–100 nM) for 24 hours or 10 nM BPR0L075 for indicated durations (0–48 hours), stained with propidium iodide, and analyzed by flow cytometer. The cell cycle profiles are presented as three-dimensional overlay. The X-axis shows the intensity of propidium iodide fluorescence, which indicates cellular DNA content in different cell cycle phases. The Y-axis represents the cell counts; and z-axis shows the concentration or time points of BPR0L075 treatment. 2N, cells residing in the G0–G1 phase of cell cycle; 4N, cells in the G2 phase or mitosis. BPR0L075 induces significant G2/M arrest followed by appearance of a sub-G1 population in both parental and resistant cells, with polyploid cells (arrows) only in the resistant cells. Results are representative of three independent experiments.

We followed the cell cycle progression in both parental and paclitaxel-resistant cells after BPR0L075 treatment. As shown in Figure 3, BPR0L075 treatment (10–100 nM, drug levels can be achieved in mouse xenografts [22]) for 24 hours resulted in losses of cell population from G0-G1 phases, with concomitant accumulation of cells in the G2/M phase in both OVCAR-3 and OVCAR-3-TR cells. In comparison to the robust cell cycle arrest by BPR0L075, paclitaxel-resistant cells exhibited defective mitotic response to paclitaxel, which failed to arrest in mitosis even treated with 100 nM of paclitaxel (data not shown). For OVCAR-3-TR cells, polyploid cells (>4N DNA) appeared after BPR0L075 treatment comparing with OVCAR-3 cells. We also investigated the time course of BPR0L075 induced cell cycle arrest when treated with 10 nM BPR0L075. BPR0L075 started to block the OVCAR-3 cells at G2/M phase as early as 12 hour, and progressed to complete arrest from 24 to 48 hours in both OVCAR-3 and OVCAR-3-TR cells. BPR0L075 treated OVCAR-3-TR cells gave rise to a significant fraction of polyploid cells (DNA content >4N) at 24–48 hours (Figure 3, arrows). Similarly, BPR0L075 treatment also induced concentration- and time-dependent cell cycle arrest in the G2/M phase in both the SKOV-3 and SKOV-3-TR cells, with high fraction of polyploid cells in SKOV-3-TR (Figure S1). Mitotic cell death or mitotic catastrophe is often associated with growth arrest in the G2/M phase of the cell cycle followed by the formation of large polyploid cells [31], [32], these polyploid cells observed suggests that BPR0L075 induced mitotic catastrophe in paclitaxel-resistant ovarian cancer cells.

### BPR0L075 Disrupts the Microtubule Cytoskeleton and Induces Mitotic Catastrophe in Paclitaxel-resistant Ovarian Cancer Cells

**Figure 4:**
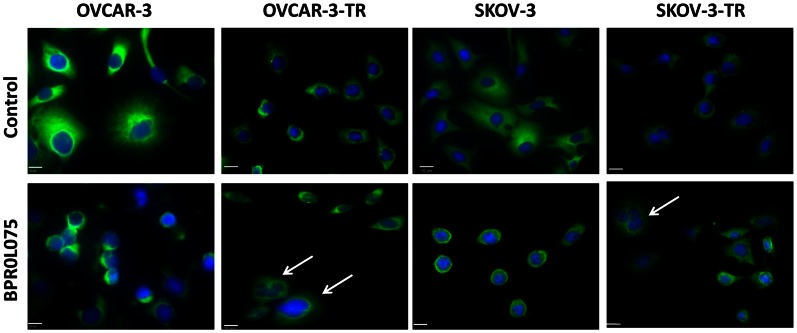
BPR0L075 treatment disrupts microtubule networks in parental and paclitaxel-resistant ovarian cancer cells. Coverslips containing OVCAR-3 and SKOV-3 parental and paclitaxel-resistant cells were exposed to medium containing ethanol vehicle or 20 nM of BPR0L075 for 18 hours. The cells were fixed, coverslips were then stained with anti-α-tubulin antibody (AlexaFluor 488, green) for microtubules and 4,6-diamidino-2-phenylindole (DAPI, blue) for cell nuclei, and observed by Olympus IX81 fluorescence microscope. The control parental cells showed typical radial microtubule arrays. BPR0L075 treatment disrupted microtubule cytoskeleton in both the parental and resistant ovarian cancer cells. White arrows show the giant, multinucleated cells characteristic of mitotic catastrophe. Scale bar, 10 µm.

We examined the effect of BPR0L075 on the organization of microtubule networks of ovarian cancer cells by immunofluorescence staining of α-tubulin (green). As shown in Figure 4, ethanol vehicle treated OVCAR-3 and SKOV-3 cells had well organized typical radial microtubule network arrays; in contrast, BPR0L075 (20 nM for 18 hours) treated OVCAR-3 and SKOV-3 cells had rounded shape, condensed chromosomes, with the disruption of the microtubule fibers of the cells that accompanied by cellular deformation. For the paclitaxel-resistant OVCAR-3-TR and SKOV-3-TR cells, the long microtubule fibers were rarely seen in vehicle treated cells, with even less staining after treatment of BPR0L075. BPR0L075 treated resistant cells also showed characteristics of mitotic catastrophe as defined by the formation of giant, multinucleated cells (Figure 4, arrows).

### Paclitaxel-resistant Ovarian Cancer Cells Display Deregulated Mitotic Response and Depletion of Surviving

**Figure 5:**
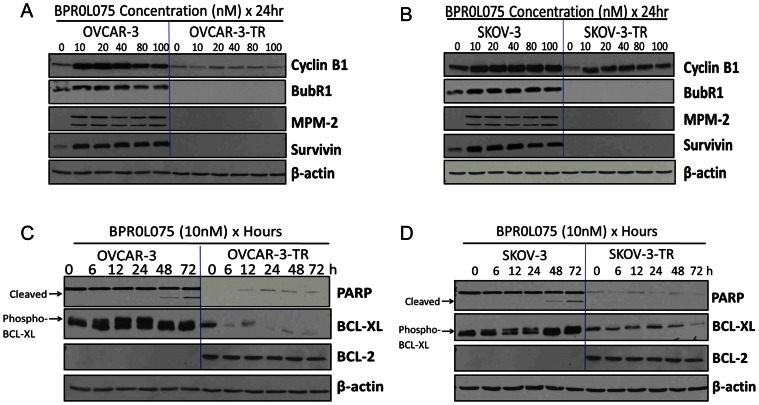
Western blot analysis of the relative protein expressions in the parental and paclitaxel-resistant ovarian cancer cell lines after BPR0L075 treatment. BPR0L075 treatment (10–100 nM for 24 hours) induced changes in cyclin B1, BubR1, MPM-2, and survivin expressions in OVCAR-3/OVCAR-3-TR cells (A) and SKOV-3/SKOV-3-TR cells (B). BPR0L075 treatment (10 nM) induced changes in PARP, BCL-XL, and BCl-2 proteins at different time (0–72 hours) in OVCAR-3/OVCAR-3-TR cells (C) and SKOV-3/SKOV-3-TR cells (D). 50 µg of the protein lysate was loaded on the SDS-PAGE and experiments were repeated and representative blots are shown.

We analyzed the key molecular events associated with BPR0L075 induced cell cycle arrest. Cyclin B1 is a cell cycle regulatory protein involved in mitosis and expressed predominantly during G2/M phase of cell cycle. Western blot analysis showed that the basal expressions of cyclin B1 in paclitaxel-resistant OVCAR-3-TR and SKOV-3-TR cells were significantly lower than those in OVCAR-3 and SKOV-3 cells. BPR0L075 treatment (10–100 nM for 24 hours) caused a concentration-dependent increase in cyclin B1 levels in both parental cell lines and paclitaxel-resistant sublines (Figure 5A, B). BPR0L075 treatment also induced up-regulation of mitotic spindle checkpoint protein BubR1 in parental OVCAR-3 and SKOV-3 cells. In paclitaxel-resistant OVCAR-3-TR and SKOV-3-TR cells, the endogenous expression of BubR1 became undetectable (Figure 5A, B). We also monitored the MPM-2 protein, an established marker of mitotic cells that recognizes a group of phosphorylated forms of proteins that are phosphorylated only in mitosis [33], [34]. BPR0L075 treatment (10–100 nM for 24 hours) caused immediate induction of MPM-2 in parental cells, but not in the resistant sublines that have mitotic defects (Figure 5A, B). We also observed the endogenous expression of survivin, a mitosis regulator and inhibitor of apoptosis protein, in OVCAR-3 and SKOV-3 cells (Figure 5A, B). BPR0L075 mediated mitotic arrest is associated with the induction of survivin in a concentration-dependent manner in parental cells, which preserves a survival pathway for cancer cells. In contrast, the survivin expression was depleted in the OVCAR-3-TR and SKOV-3-TR sublines, BPR0L075 treatment failed to induce survivin up-regulation in the paclitaxel-resistant cells (Figure 5A, B).

### BPR0L075 Induces Phosphorylation of Bcl-XL in Parental Cells, but not in Paclitaxel-resistant Ovarian Cancer Cells

Bcl-2 family proteins are key regulators of apoptosis, and their expression levels have been suggested to correlate with sensitivity to paclitaxel [35], [36], [37]. The OVCAR-3 and SKOV-3 cell lines did not express Bcl-2 protein to an immunodetectable level, but were found to express high levels of Bcl-XL. In contrast, for the OVCAR-3-TR and SKOV-3-TR sublines, the Bcl-2 protein level was easily detectable which associated with decreased expression of Bcl-XL (Figure 5C, D). When treated the parental cells with 10 nM BPR0L075, the phosphorylated form of Bcl-XL appeared at 12–72 hours, evidenced by the electrophoretic mobility shifts on immunoblots (Figure 5C, D). However, similar exposure of BPR0L075 did not induce Bcl-2 or Bcl-XL phosphorylation in OVCAR-3-TR and SKOV-3-TR sublines. BPR0L075 treatment reduced Bcl-XL levels in OVCAR-3-TR and SKOV-3-TR cells without affecting the Bcl-2 levels (Figure 5C, D).

### BPR0L075 Induced Cell Death is Caspase-3 Independent in Both the Parental and Paclitaxel-resistant Ovarian Cancer Cells

**Figure 6:**
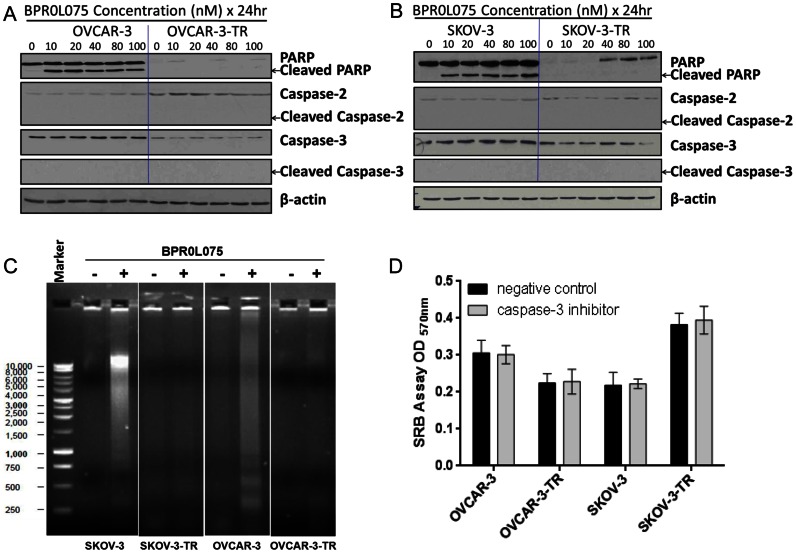
BPR0L075 induces PARP cleavage and DNA fragmentation in parental cells, but not in the paclitaxel-resistant ovarian cancer cells. Western blot analysis of the PARP, caspase-2, and caspase-3 after BPR0L075 (10–100 nM) treatment for 24 hours in the OVCAR-3/OVCAR-3-TR cells (A) and SKOV-3/SKOV-3-TR cells (B). β-actin was loading control. (C) DNA ladder assays in cells treated with 10 nM BPR0L075 for 72 hours. Genomic DNA was subjected to 1% agarose gel electrophoresis. The gel was stained with ethidium bromide and the DNA bands were visualized under an ultraviolet transilluminator. (D) Effect of caspase-3 inhibitor on BPR0L075 induced cytotoxicity. Cells were pretreated with caspase-3 inhibitor (20 µM Z-DEVD-FMK) or negative control inhibitor (20 µM Z-FA-FMK) for 24 hours before incubation with 10 nM BPR0L075 for additional 72 hours. On completion of the incubation, the cell viability was determined by SRB assay. No statistically significant difference of cytotoxicity was observed for each pair of cell lines (*P*>0.05, student *t*-test). Data represent mean ± SD.

BPR0L075 treatment induced proteolytic cleavage of poly(ADP-ribose) polymerase (PARP) from a MW of 116 KDa polypeptide to a MW of 85 KDa fragment in OVCAR-3 and SKOV-3 cells in a time- (Figure 5C, D) and concentration- (Figure 6A, B) dependent manner. However, PARP cleavage was not observed in the OVCAR-3-TR and SKOV-3-TR cells (Figure 5C, D, 6A, B), despite the fact that the BPR0L075 treatment killed both parental and resistant cells with similar IC_50_ values (Figure 1). We also observed that BPR0L075 treatment induced DNA ladder formation, a hallmark of cell apoptosis, in the SKOV-3 and OVCAR-3 cells, but not in the SKOV-3-TR and OVCAR-3-TR sublines (Figure 6C). Using western blot analysis, we observed no activation of caspase-3 (loss of procaspase 3 or appearance of the cleaved caspase-3) in both parental and resistant cells after BPR0L075 treatment using either antibodies specific recognized procaspase-3 or cleaved caspase-3 (Figure 6A, B). To further confirm a caspase-3 independent cell death pathway, we pretreated the cells with a caspase-3 specific and irreversible inhibitor (20 µM Z-DEVD-FMK) or negative control inhibitor (20 µM Z-FA-FMK) 24 hours before the addition of BPR0L075. No statistically significant difference of cytotoxicity was observed for each pair of cell lines (Figure 6D, *P*>0.05, student *t*-test); caspase-3 inhibitor did not prevent cell death in both the parental and resistant cells. Mitotic catastrophe is known to result in cell death by caspase-2 and caspase-3 dependent and independent mechanisms [38]. We checked the caspase-2 activation after treatment with BPR0L075, caspase-2 cleavage was not detected in both parental and resistant cells (Figure 6A, B). Together, the data indicate that caspase-2 and caspase-3 independent cell death was involved in both the parental and paclitaxel-resistant ovarian cancer cells; the predominant cell death pathway was through apoptosis in parental cells, and mitotic catastrophe in resistant ovarian cancer cells.

## Discussion

Paclitaxel is one of the most successful chemotherapeutic agents and used for the treatment of a wide variety of human malignancies, including solid tumors and hematological malignancies [4], [39]. Paclitaxel is known to bind to β-tubulin and promote its assembly into microtubules [40], which leads to cell cycle arrest at the G2/M phase, activates mitotic spindle “checkpoint”, and eventually leads to apoptotic cell death [4]. Paclitaxel is a front-line agent for ovarian cancer chemotherapy, along with the platinum agents. However, the treatment of advanced ovarian cancer with paclitaxel is hindered by the development of drug resistance. Identifying novel agents that overcome paclitaxel resistance is urgently needed. BPR0L075 is a heterocyclic 3-aroylindole microtubule-destabilizing agent, which interferes with tumor cell microtubule dynamics, leads to cancer cell cycle arrest and cell death by caspase-3 mediated apoptosis [20] and antiangiogenic effects [21]. In addition, BPR0L075 disrupts tumor vasculature and shuts down tumor blood perfusion *in vivo* [22]. In the current study, we tested the effectiveness and mechanism of BPR0L075 in treating the paclitaxel-resistant ovarian cancer cells.

Our results show that ovarian cancer cell lines that resistant to microtubule-stabilizing agent paclitaxel may remain sensitive to microtubule-destabilizing agent BPR0L075. BPR0L075 displayed potent cytotoxicity in both parental ovarian cancer cells and their paclitaxel-resistant sublines with IC_50_ values around 5 nM, while equal concentration of paclitaxel treatment was ineffective in highly resistant ovarian cancer cells (Figure 1). These paclitaxel-resistant ovarian cancer cells also showed cross- resistance to other chemotherapeutics such as doxorubicin (Figure 1B). This observation is consistent with the prior report that BPR0L075 is effective against multidrug-resistant human cervical carcinoma KB cells that are resistant to vincristine, paclitaxel, and colchicine [20]. Our results indicate that BPR0L075 is a potential useful agent in controlling multidrug-resistant ovarian cancer cells.

Our data show that BPR0L075 overcomes two main mechanisms of resistance to paclitaxel, i.e., MDR1 resistance attributable to P-glycoprotein overexpression and alteration of the class III β-tubulin isotype expression. The paclitaxel-resistant OVCAR-3-TR and SKOV-3-TR sublines showed overexpression of P-gp (Figure 2A), an efflux pump able to hamper retention of taxanes and other cationic drugs [41]. BPR0L075 is not a substrate for the P-gp and BCRP efflux pumps, evidenced by the similar BPR0L075 intracellular levels achieved in the parental and resistant sublines (Figure 2C) and comparable IC_50_ values in MCF7 and MCF7/MX cells. Both OVCAR-3 and SKOV-3 cells have high expression of class III β-tubulin, while the OVCAR-TR and SKOV-3-TR showed markedly diminished expression of class III β-tubulin (Figure 2A), which suggests that the alteration of class III β-tubulin expression affects the sensitivity to paclitaxel but not BPR0L075. BPR0L075 is also highly effective in killing 1A9-PTX10 cells with β-tubulin gene mutation [27], [28] (Figure 1). Our results support the previous reports that mutations in β-tubulin and alterations in the expression of class III β-tubulin isotypes induce resistance to microtubule-stabilizing agents and hypersensitivity to microtubule-destabilizing agents through an alteration in the microtubule assembly and dynamics in cancer cells [42]. Together, our results suggest that paclitaxel and BPR0L075 bind at different sites of tubulin; have non-overlapping mechanism of resistance, thus potentially allowing the use of BPR0L075 for those ovarian cancer patients relapsing after paclitaxel based chemotherapy.

BPR0L075 induced cell cycle arrest at G2/M phase in the parental and paclitaxel-resistant ovarian cancer cells when paclitaxel treatment was virtually ineffective (Figure 3, Figure S1). The flow cytometry data show that BPR0L075 does not cause complete mitotic arrest in paclitaxel-resistant cells, indicating that the resistant cells may still undergo DNA synthesis despite failure of cell division. BPR0L075 treated resistant cells gave rise to a significant fraction of polyploid cells (Figure 3, Figure S1), suggesting that BPR0L075 induced cell death in paclitaxel-resistant cells proceed through mitotic catastrophe. Mitotic catastrophe is another form of cell death pathway that is caused by abnormal mitosis and/or irreparable chromosomal damage [32], [43], [44], [45]. Mitotic catastrophe occurs during or shortly after a deregulated or failed mitosis and can be accompanied by morphological alteration such as micronucleation (the appearance of chromosomes or chromosomal material outside of the two daughter nuclei) and multinucleation (the appearance of two or more nuclei similar or heterogeneous in size, resulting from deficient separation during cytokinesis) [43], [44], [45]. The formation of giant multinucleated cells is a phenotype of mitotic catastrophe in cells induced by ionizing radiation [46], [47] or anticancer drugs influencing microtubule stability [43], [48], [49], [50]. Immunofluorescence staining shows the appearance of giant, multinucleated cells (Figure 4) following BPR0L075 treatment of the resistant cells, suggesting that chromosome segregation do occur at least to some extent in paclitaxel-resistant cells but cytokinesis failed.

Our study shows that mitotic response pathways are highly deregulated in the OVCAR-3-TR and SKOV-3-TR cancer cells, evidenced by down-regulation of cyclin B1, depletion of mitotic spindle checkpoint protein BubR1, and inability to activate mitotic epitope MPM-2 in resistant cells when compared with the parental cells (Figure 5A, B). The observations are consistent with reports that decreased expression of cyclin B1 and BubR1 is associated with weakened spindle checkpoint and paclitaxel resistance in ovarian carcinoma cells [51]; depletion of BubR1 with siRNA resulted in loss of spindle assembly checkpoint function and resistance to paclitaxel [52].

Survivin depletion in paclitaxel-resistant ovarian cancer cells could play a role in the BPR0L075-induced mitotic catastrophe. Survivin is a bifunctional protein that acts as a suppressor of apoptosis and a mitotic regulator [53], [54], [55]. Survivin inhibits apoptosis by interacting with caspase and Smac/DIABLO proteins, suppress the activation of downstream caspases, which preserves a survival pathway [56]. Survivin also modulates several mitotic events, including spindle and interphase microtubule organization, the spindle assemble checkpoint and cytokinesis [44]. We observed the loss of basal expression of survivin in paclitaxel-resistant cells when compared with parental cells (Figure 5A, B), suggesting the weakening of spindle checkpoint in the resistant cells. Paclitaxel is known to be ineffective in treating the survivin-depleted cancer cells [57], [58]. It was reported that reduction or loss of survivin is associated with several mitotic defects, including hyperduplication of centrosomes and aberrant spindle assembly [44]. Mammalian cells lacking survivin are unable to align their chromosomes and become polyploid at a very high frequency [58]. Recently, it was reported that knocking down of survivin using siRNA can enhance sensitivity to BPR0L075 in human cervical carcinoma KB cells and cause mitotic cell death; overexpression of survivin counteracts the therapeutic effect of BPR0L075 [42]. Additionally, it has been reported that knock-down of survivin using siRNA causes cell death via mitotic catastrophe in neuroblastoma [59], hepatocellular carcinoma [60], and gastric cancer [61]. Survivin inhibition using antisense oligo results in mitotic catastrophe in human neural tumor cells [62]. Together, the depletion of survivin and deregulated mitotic checkpoint in the resistant cells could potentially contribute to BPR0L075 induced mitotic catastrophe.

It was reported that changes in apoptotic regulatory proteins such as Bcl-2 and Bcl-XL played important roles in the paclitaxel resistance [35], [36], [37]. We observed that parental ovarian cancer cells have undetectable level of Bcl-2 and exhibited phosphorylation of Bcl-XL after BPR0L075 exposure. In contrast, paclitaxel-resistant ovarian cancer cells displayed increased Bcl-2 expression, but failed to induce phosphorylation of Bcl-XL or Bcl-2 after BPR0L075 exposure. BPR0L075 treatment induced down-regulation of Bcl-XL without changing of Bcl-2 levels in resistant cells (Figure 5C, D). Bcl-2 family members usually substitute for each other, for example, cells expressing low Bcl-2 levels express high levels of Bcl-XL [35]. Previous study has reported that the posttranslational Bcl-XL and Bcl-2 phosphorylation in malignant cells after incubation cells with microtubule disruption agents including paclitaxel, vincristine, vinblastine, colchicine, nocodazole, epothilones B [35]. Our study showed that microtubule-depolymerizing agent BPR0L075 is also capable of inducing Bcl-XL phosphorylation in parental ovarian cancer cells, but not in the paclitaxel-resistant cells, where mitotic catastrophe is the dominant mode of cell death. This is consistent with a previous report that Bcl-2 inhibits etoposide-induced apoptosis, however, Bcl-2 has no effect on the formation of multinucleated cells characteristic of mitotic catastrophe [48]. Together, our results suggest that BPR0L075 induced mitotic catastrophe can bypass apoptosis-resistance mechanism and changes in apoptotic regulatory proteins such as Bcl-2 and Bcl-XL.

Our results show for the first time that BPR0L075 can induce cell death by a dual mechanism in parental and paclitaxel-resistant ovarian cancer cells. In the parental cells, BPR0L075 treatment resulted in apoptotic biochemical features in dying cells, such as cleavage of PARP and internucleosomal DNA fragmentation formation, a hallmark of apoptosis (Figure 6). In contrast, in the paclitaxel-resistant cells, BPR0L075 induced cell death proceeded without the cleavage of PARP and DNA ladder formation (Figure 6), instead, BPR0L075 induced mitotic catastrophe, resulted in formation of large polyploid cells (Figure 3, 4). Our results are consistent with previous reports that cells that die through mitotic catastrophe usually do not show DNA ladder formation or DNA breaks that are detectable by TUNEL staining [44], [63], [64], [65].

Our data also show that BPR0L075 kills cells through caspase-3 independent pathways in both the parental and the paclitaxel-resistant ovarian cancer cells, evidenced by the absence of cleaved caspase-3 protein (Figure 6A, B) and ineffectiveness of caspase-3 inhibitor in protecting both the parental and resistant cells from BPR0L075 induced cell death (Figure 6D). In parental ovarian cancer cells, survivin is up-regulated after BPR0L075 treatment; caspase-3 activation could be suppressed by survivin, which functions a counteraction protein against caspases. In the paclitaxel-resistant cells, mitotic catastrophe is the major cell death pathway; it has reported that mitotic catastrophe can proceed via caspase-3 dependent or independent pathways [44], [66]. BPR0L075 was previously reported to induce apoptosis by activation of caspase-3 in human cervical carcinoma KB cells [20]. Recently a study showed that BPR0L075 killed human colorectal cancer cells HCT116 through a caspase-independent mechanism involving activation of JNK and p38 MAPK pathways [67]. Our results support a caspase-3 independent pathway in both the parental and the paclitaxel-resistant ovarian cancer cells. This discrepancy could be tumor cell type dependent. Mitotic catastrophe may be mediated in a caspase-2 dependent or independent fashion [38]. Our results show no activation of caspase-2 in BPR0L075 induced mitotitc catastrophe in resistant cells. It has reported that combrestatin CA-4 induced caspase-2 independent mitotic catastrophe in non-small cell lung cancer cells [68]. Cisplatin treatment resulted in caspase-2 independent mitotic castrophe in p53 non-functional ovarian cancer cell lines [69]. Together, this indicates that caspase-2 activation is not a requirement for mitotic catastrophe.

In conclusion, we have shown that tubulin-depolymerizing agent BPR0L075 overcomes two main mechanisms of resistance to paclitaxel that includes up-regulation of membrane efflux transporters and alterations in βIII-tubulin isoform expression. When the paclitaxel-resistant ovarian cancer cells fail to respond to the apoptosis inducing chemotherapeutics such as taxane and doxorubicin, they still arrest in mitosis after tubulin-depolymerizing agent BPR0L075 treatment at low nanomolar concentration. The mitotic catastrophe could be the major mechanism of BPR0L075 induced cell death in the paclitaxel-resistant ovarian cancer cell lines SKOV-3-TR and OVCAR-3-TR, which have depleted survivin and deregulated mitotic checkpoint. Mitotic catastrophe in our models is independent of caspase-2 and caspase-3 activation. At present, few conventional anticancer treatments can overcome multidrug resistance by inducing cell death via mitotic catastrophe. BPR0L075 can induce cell death by a mechanism that is not reliant on apoptosis induction, and thus representing a promising microtubule therapeutic for the treatment of multidrug-resistant ovarian cancer.

## Materials and Methods

### Chemicals

The compound BPR0L075 was synthesized as described previously [70]. BPR0L075 is white solid and dissolved in ethanol as 2 mM stock solution. DAPI (4, 6-diamidino-2-phenylindole) was obtained from Invitrogen (Carlsbad, CA). Paclitaxel, doxorubicin, 1-(1H-indol-3-ylcarbonyl)-1H-imidazole, and all other chemicals were purchased from Sigma-Aldrich (St. Louis, MO) and were of standard analytic grade or higher.

### Cell Culture

Human ovarian cancer cell lines SKOV-3 and OVCAR-3 were obtained from American Type Culture Collection (ATCC, Manassas, VA). Paclitaxel-resistant clones (SKOV-3-TR and OVCAR-3-TR) were selected by continuous treatment with paclitaxel over six months [71]. OVCAR-3 cells were maintained in RPMI-1640 medium (Irvine Scientific, Santa Ana, CA) supplemented with 10% fetal bovine serum (FBS; Gemini Bio-Products, Inc., Calabasas, CA); OVCAR-3-TR were maintained in the same medium containing 0.5 µM of paclitaxel. SKOV-3 cells were grown in McCoy’s 5A medium supplied with 10% FBS; SKOV-3-TR cells were maintained in the same medium containing 0.25 µM of paclitaxel. Ovarian cancer cell line A2780-1A9 and its paclitaxel resistant subline 1A9-PTX10 were obtained from Dr. Tito Fojo (National Cancer Institute, Bethesda, MD) [29]. A2780-1A9 cells were cultured in RPMI-1640 medium with 10% FBS, the 1A9-PTX10 cells were maintained in the same medium supplemented with 15 ng/mL paclitaxel and 5 µg/mL verapamil (a P-gp antagonist) [10], [29]. All resistant cells were grown in paclitaxel-free, verapamil-free medium for at least two days prior to any assays. Human breast cancer cell line MCF7 was obtained from ATCC and its mitoxantrone-resistant subline MCF7/MX was cultured in complete RMPI medium in the presence of 250 nM mitoxantrone as described [72]. All cells were grown in incubator at 37°C in humidified 95% air and 5% CO_2._

### In vitro Cytotoxicity Assay

All cytotoxicity assays were performed in 96-well plates using a sulforhodamine B (SRB) colorimetric assay. Cells were plated at 1000–2500 cells/well in 100 µL of complete medium in 96-well plates. After overnight incubation to allow attachment, a stock solution of BPR0L075 or paclitaxel (2 µM in ethanol) was diluted to the desired concentrations in 100 µL of medium immediately before each experiment. The final drug concentrations ranged from 3–15 nM after addition of BPR0L075 or paclitaxel containing medium in replicates of 12 wells per condition. The final concentration of doxorubicin ranged from 0.1–1000 nM. Control wells received ethanol in whole medium equivalent to the maximum final ethanol concentration of drug treated wells. The cells were also pretreated with 20 µM caspase-3 inhibitor Z-DEVD-FMK or negative control inhibitor Z-FA-FMK (BD Pharmingen) for 24 hours before addition of 10 nM of BPR0L075. After incubation of the cells with drugs for 4 days, cell monolayers are fixed with 10% (wt/vol) trichloroacetic acid and stained with sulforhodamine B solution for 30 min, after which the excess dye is removed by washing repeatedly with 1% (vol/vol) acetic acid. The protein-bound dye is dissolved in 10 mM Tris base solution for optical density determination at 570 nm using a microplate reader (BioTek Instrument, Inc.). The IC_50_ values resulting from 50% inhibition of cell growth were calculated graphically from fitted median effect plot using the CompuSyn software (Biosoft, Cambridge, UK). The fold of drug resistance was calculated from the ratio of the IC_50_ of the resistant and parental cells.

### Western Blot Analysis

Cells were lysed at 4°C in RIPA buffer (150 mM NaCl, 10 mM Tris, pH 7.4, 0.1% SDS, 1.0% Triton X-100, 5 mM EDTA, Boston BioProducts Inc.) The protein concentration of cell lysates was determined by bicinchoninic acid (BCA) assay (Pierce, Rockford, IL). Proteins were resolved on 7–12% Tri-glycine gels and transferred onto nitrocellulose membranes. Blots were probed with primary antibodies and visualized with enhanced chemiluminescence detection reagent (Pierce). Anti-α-tubulin antibody (Sigma) was used for tubulin staining, anti-phosphorylated Ser/Thr antibody MPM-2 (Millipore) was used for detecting mitotic cells; antibodies against survivin, cyclin B1, BubR1, PARP, caspase-2, caspase-3, cleaved caspase-3, Bcl-2, Bcl-XL, β-tubulin, β-actin were purchased from cell signaling. Anti-class III β-tubulin antibody was obtained from Promega, anti-P-glycoprotein mAb (C219) was purchased from Calbiochem. Blots were usually stripped with re-stripping buffer (Pierce) and re-probed with β-actin antibody for equal loading control.

### Intracellular BPR0L075 Quantification by LC/MS/MS

Exponentially growing cells (2×10^6^) were plated in 60 mm petri dish with 5 mL of complete medium. After overnight incubation to allow cell attachment, medium was replaced with freshly prepared complete medium containing 20 nM BPR0L075. After 6 hours of incubation, the cells were washed with ice-cold PBS three times, and harvested. Cell pellets were resuspended in 100 µL of RIPA solubilizing buffer and disrupted by vigorously vortexing for 1 min, then 50 µL of cell lysate containing 150 µg of proteins was used for LC/MS/MS analysis. 1-(1H-indol-3-ylcarbonyl)-1H-imidazole was employed as an internal standard (IS). Proteins were precipitated by adding 140 µL of chilled acetonitrile with sonication and centrifugation at 12,000 rpm for 10 min, then 20 µL of supernatants were injected into the Varian 1200 LC system coupled with a triple quadrupole mass spectrometer with an electrospray ionization source in the positive ion mode (Palo Alto, CA). The chromatographic separation was performed on a Varian C18 column (150 mm×2.0 mm, 5 µm particle size) at ambient temperature. Isocratic elution with acetonitrile (0.5% formic acid): water (0.5% formic acid) (70∶30, v/v) was delivered at a flow rate of 0.2 mL/min. Argon was the collision gas. Selected reaction monitoring (SRM) of the precursor-product ion transitions m/z 342 → 195 for BPR0L075, and 212→ 144 for IS were used for quantification. The calibration curve was generated by spiking 10 µL of BPR0L075 and IS (10 µM stock) in 50 µL of blank cell lysates to achieve a BPR0L075 concentration of 10–2000 nM. The intracellular concentration of BPR0L075 was expressed as pmol of BPR0L075/mg of protein.

### Cell Cycle Analysis

Cell cycle distribution was evaluated by BD FACSCalibur (Becton Dickinson, San Jose, CA). Briefly, cells were treated with 10–100 nM concentration of BPR0L075 for 24 hours, or 10 nM of BPR0L075 for 0, 6, 12, 24, 36, and 48 hours. Both the adherent and non-adherent cells were collected, washed with ice-cold PBS, and fixed in 70% ethanol at −20°C for at least 24 hours. Cell pellets were then washed with PBS and stained with 0.4 mL propidium iodide (5 µg/mL) in 0.1% RNase for 30 min at dark. The DNA content was analyzed based on propidium iodide fluorescence using FACSCalibur equipped with a 488 nm laser and a 610±20 nm band-pass filter. Percentage of cells in different cell cycle stages were estimated by the observed distribution of DNA content, i.e., sub-G_1_ (DNA <2N), G_0_–G_1_ (DNA = 2N), S (2N<DNA <4N), G_2_-M (DNA = 4 N), and polyploid (DNA >4N). All experiments were repeated at least three times.

### Immunofluorescence Staining

Exponentially growing cells were plated on sterile cover slips (12 mm) placed in a 12-well plate at a density of 50,000 cells per well, and cells were allowed to attach overnight. Cells were treated with ethanol control or BPR0L075 at a final concentration of 20 nM for 18 hours at 37°C. Media were removed and cells were washed with PBS, followed by fixation with ice cold acetone and methanol cosolvent (1∶1, v/v) for 5 min, washed with PBS three times. Coverslips were permeablized and blocked in PBS containing 1% goat serum and 0.25% Tween 20 for 30 min at 4°C. Blocked coverslips were incubated with a mouse monoclonal anti-α-tubulin antibody (Sigma, 1∶1000 dilution) in blocking solution. Cells were then washed with PBS before incubating with AlexaFluor 488 goat anti-mouse secondary antibody (Invitrogen, 1∶1000 dilution) at 4°C for 1 hour. Coverslips were then rinsed with PBS and incubated with 1 µg/mL DAPI in washing solution for 2 min at room temperature for nuclear recognition before they were mounted onto glass slides with FluorSave reagent (Calbiochem). Immunofluorescence was analyzed on an Olympus IX81 inverted fluorescent microscope. Excitation and emission filters were 360±20 nm and 460±25 nm for blue channel (DAPI) and 470±40 nm and 525±50 nm for green channel (Alexa Fluor 488). The morphology of multinuclei (two or more than two nuclei in one cell) was confirmed under fluorescent microscope and compared with the observations obtained under the light field.

### DNA Ladder Fragmentation Assay

Exponentially growing cells (3 million/60 mm dish) were treated with 10 nM BPR0L075 for 72 hours and harvested by trypsinization, washed with PBS, and incubated in 100 µL of lysis buffer (150 mM NaCl, 10 mM Tris, pH 7.4, 0.1% SDS, 1.0% Triton X-100, 5 mM EDTA) for 1 hour at 50°C, followed by the addition of 0.5 vol of 5 M NaCl and incubation on ice for 10 min. The lysates were then centrifuged at 15,000 rpm for 20 min at 4°C. The supernatant was collected and extracted by phenol/chloroform/isoamyl alcohol (25∶24∶1, v/v/v) (Invitrogen), and DNA was precipitated by addition of 2 vol of ice cold absolute ethanol. The DNA pellet was dissolved in 20 µL of TE buffer [10 mM Tris-HCl and 1 mM EDTA, pH 8.0] together with 1 µL of RNAse (10 µg/mL) and 1 µL of Proteinase K (100 µg/mL) and incubated at 37°C for 30 min. The purified DNA and loading dye was subjected to 1% agarose gel electrophoresis at 50 V for 10 min and 100 V for 1 hour. The gel was stained with 0.5 µg/mL ethidium bromide (Fisher) and the DNA bands were visualized under an ultraviolet transilluminator using VersaDoc 5000 (Bio-Rad).

### Statistical Analysis

The experimental values are expressed as mean ± standard deviation (SD). All statistical analysis was done using GraphPad Prism® 5 software (San Diego, CA, USA). Difference between two means of the experimental groups and the control groups were tested using unpaired two-sided Student’s *t*-test. Differences with *P*<0.05 were considered significant.

## Supporting Information

Figure S1 **BPR0L075 induces concentration- and time-dependent cell cycle arrest in both SKOV-3 and SKOV-3-TR cells.** Cells were treated with ethanol vehicle or BPR0L075 (10–100 nM) for 24 hours or 10 nM BPR0L075 for indicated durations (0–48 hours), stained with propidium iodide, and analyzed by flow cytometer. The cell cycle profiles are presented as three-dimensional overlay. The X-axis shows the intensity of propidium iodide fluorescence, which indicates cellular DNA content in different cell cycle phases. The Y-axis represents the cell counts; and z-axis shows the concentration or time points of BPR0L075 treatment. 2N, cells residing in the G0–G1 phase of cell cycle; 4N, cells in the G2 phase or mitosis. BPR0L075 induces significant G2/M arrest followed by appearance of a sub-G1 population in both parental and resistant cells, with polyploid cells (arrows) in the resistant cells. Results are representative of three independent experiments.
(TIF)
