# Supplementary figures and images for: An Antimitotic and Antivascular Agent BPR0L075 Overcomes Multidrug Resistance and Induces Mitotic Catastrophe in Paclitaxel-Resistant Ovarian Cancer Cells
Source: PLoS One. 2013 Jun 6;8(6):e65686. doi: 10.1371/journal.pone.0065686 (PMC3675084; doi:10.1371/journal.pone.0065686)

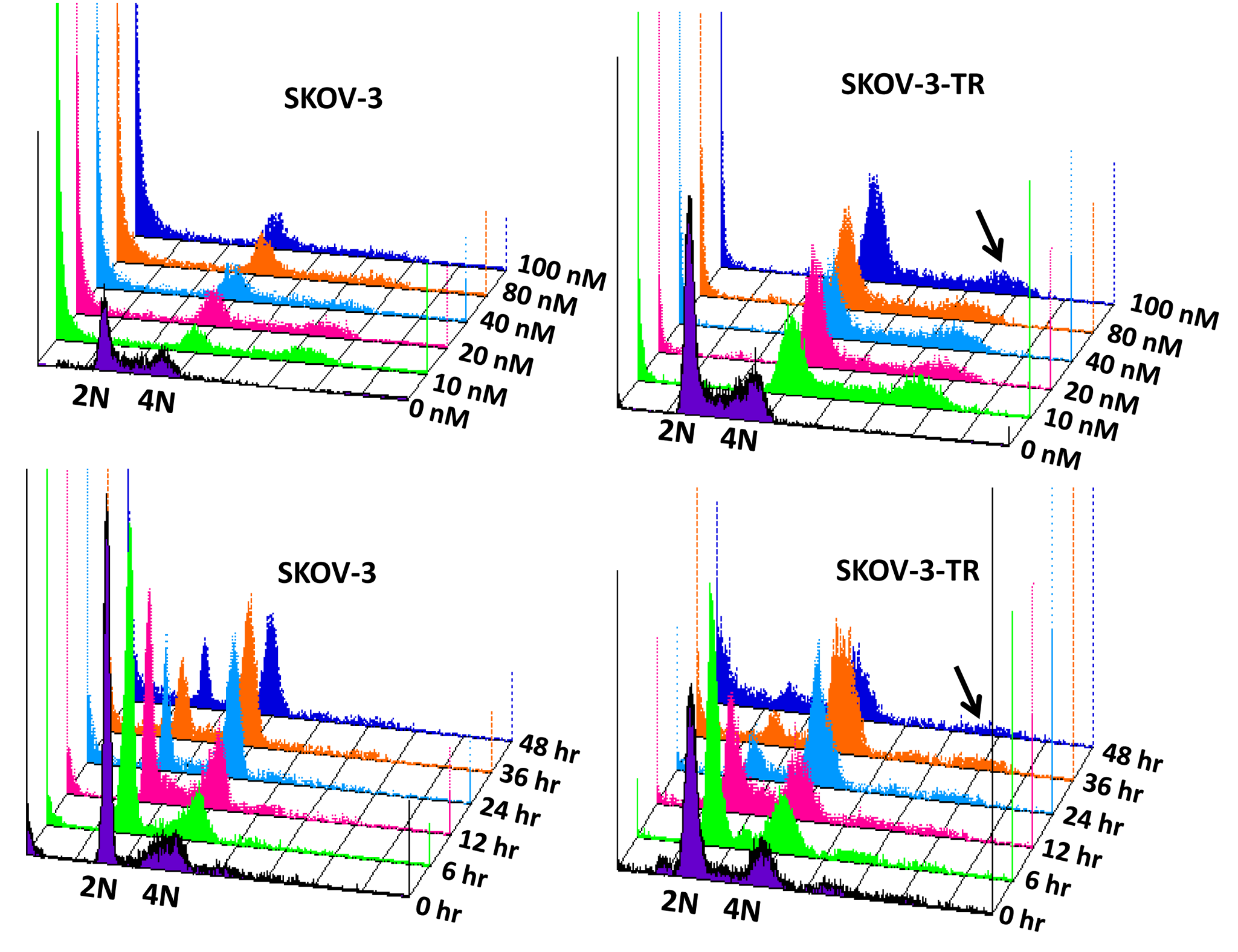

Supplement: Figure S1 — BPR0L075 induces concentration- and time-dependent cell cycle arrest in both SKOV-3 and SKOV-3-TR cells. Cells were treated with ethanol vehicle or BPR0L075 (10–100 nM) for 24 hours or 10 nM BPR0L075 for indicated durations (0–48 hours), stained with propidium iodide, and analyzed by flow cytometer. The cell cycle profiles are presented as three-dimensional overlay. The X-axis shows the intensity of propidium iodide fluorescence, which indicates cellular DNA content in different cell cycle phases. The Y-axis represents the cell counts; and z-axis shows the concentration or time points of BPR0L075 treatment. 2N, cells residing in the G0–G1 phase of cell cycle; 4N, cells in the G2 phase or mitosis. BPR0L075 induces significant G2/M arrest followed by appearance of a sub-G1 population in both parental and resistant cells, with polyploid cells (arrows) in the resistant cells. Results are representative of three independent experiments. (TIF) [file pone.0065686.s001.tif]
